# Distribution and frequency of *Vkorc1* polymorphisms in house mice and Norway rats in the northeastern United States

**DOI:** 10.1002/ps.70833

**Published:** 2026-04-21

**Authors:** Jin‐Jia Yu, Alvaro Toledo, Adrienne E Kasprowicz, Megan V Phifer‐Rixey, Xiaodan Pan, Babatunji Daramola, Changlu Wang

**Affiliations:** ^1^ Department of Entomology Rutgers University New Brunswick NJ USA; ^2^ Department of Biology Drexel University Philadelphia PA USA

**Keywords:** anticoagulant rodenticides, commensal rodents, resistance, single nucleotide polymorphisms

## Abstract

**BACKGROUND:**

Modern commensal rodent pest control often relies on anticoagulant rodenticides (ARs), yet the development of resistance driven by mutations in the *Vkorc1* gene has become a growing challenge in rodent management. Surveying the mutations in the *Vkorc1* gene in rodents is essential to understanding the rodenticide resistance profile and its impacts on the environment. This study aimed to assess the distribution and frequency of *Vkorc1* polymorphisms in house mice and Norway rats in New York, New Jersey, Pennsylvania, and Washington, DC, USA.

**RESULTS:**

A total of 147 house mice (*Mus musculus domesticus* Schwarz and Schwarz) and 143 Norway rats (*Rattus norvegicus* [Berkenhout]) were sampled from urban areas. Five nonsynonymous single nucleotide polymorphisms (nsSNPs) were identified in house mice, including A32V, W59L, L128S, Y139C, and Y139F. The A32V and Y139F mutations were reported for the first time in *M. musculus domesticus*. Among all mice examined, 84% carried at least one mutation, with Y139C (42%) and L128S (33%) being the most prevalent. At least 69% of the mice carried mutations that are known to confer resistance. In Norway rats, 35% carried mutations including two synonymous mutations (H68H, I82I) that have been previously reported and one nsSNP (L128V) that was newly identified in this study. However, H68H is not associated with AR resistance and whether the other two mutations contribute to AR resistance remains unknown.

**CONCLUSION:**

The high prevalence of resistance‐associated mutations in house mice suggests that different types of rodenticides should be alternated and integrated management approaches need to be emphasized in rodent control. © 2026 The Author(s). *Pest Management Science* published by John Wiley & Sons Ltd on behalf of Society of Chemical Industry.

## Graphical abstract

This study assesses the distribution and frequency of *Vkorc1* polymorphisms in house mice and Norway rats in northeastern USA. House mice showed higher frequency of *Vkorc1* mutations than Norway rats. The A32V and Y139F mutations are first reported in mice, and the L128V is newly reported in Norway rats.

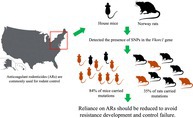

## 1 INTRODUCTION

The house mouse (*Mus musculus* L.) and the Norway rat (*Rattus norvegicus* [Berkenhout]) are globally distributed commensal rodent species. They cause substantial economic losses by damaging furniture and buildings and pose serious public health risks through the transmission of zoonotic diseases.^1^ Data from the American Housing Survey in the United States indicated that an average of 12% of occupied households experienced rodent sightings between 2018 and 2019 (U.S. Census Bureau, https://www.census.gov/data/experimental‐data‐products/small‐area‐rodent‐signs.html). In that survey, several major metropolitan areas reported higher frequencies, including Philadelphia, PA (29%), Washington, DC (20%), and Manhattan, NY (15%).

In the U.S., common rodent control strategies adopted by pest management professionals (PMPs) include sealing potential entry points to prevent infestations, setting mechanical traps, and installing bait stations loaded with rodenticides in or around structures. Among them, rodenticide baits containing first‐generation anticoagulants (FGARs) or second‐generation anticoagulants (SGARs) remain the most popular strategy due to their high efficacy and convenience of application.^2^, ^3^, ^4^ FGARs were developed in the early 1950s, followed by the introduction of SGARs in the 1970s. SGARs were much more potent than FGARs and can partially overcome resistance to FGARs.^5^ Commonly used FGARs include chlorophacinone, diphacinone, and warfarin, and widely used SGARs include brodifacoum, bromadiolone, difenacoum, and difethialone (U.S. EPA, https://www.epa.gov/rodenticides/restrictions-rodenticide-products#types).

Anticoagulant rodenticides (ARs) inhibit the vitamin K 2,3‐epoxide reductase (*Vkorc*), an enzyme essential for the vitamin K cycle, thereby disrupting the blood coagulation process.^6^ As a result, ARs cause internal hemorrhaging in rodents, ultimately leading to death. However, prolonged and widespread use of SGARs in rodent control has raised concerns about resistance development^7^, ^8^, ^9^ and their toxic effects on non‐target wildlife through primary or secondary exposure.^10^, ^11^ Furthermore, environmental contamination (e.g., soil and water) is another consequence of the application of ARs.^12^

The first observation of FGAR resistance in rodents was documented in the UK at the end of the 1950s,^13^ whereas FGAR resistance was first reported in the U.S. in the 1970s.^14^ Several mechanisms have been documented to contribute to AR resistance, including behavioral avoidance, mutations in the target gene, upregulated activity of cytochrome P450, and others.^5^ Among these mechanisms, detecting mutations in the gene encoding the vitamin K epoxide reductase complex subunit 1 (*Vkorc1*) is the most widely used approach to profiling resistance distribution in rodent populations. Single Nucleotide Polymorphisms (SNPs) in the *Vkorc1* gene have been linked to resistance against FGARs, such as warfarin, and some SGARs, including bromadiolone and difenacoum.^15^, ^16^, ^17^, ^18^ The SNPs result in amino acid substitutions that alter the conformation of the *Vkorc1* protein. This modification may decrease the binding affinity between *Vkorc* and ARs, thereby conferring resistance to FGARs and SGARs.^15^ Previous studies have documented multiple nonsynonymous mutations (nsSNPs) and synonymous mutations (sSNPs) in the *Vkorc1* gene in both house mice and Norway rats.

In house mice, the most frequently reported and widespread nsSNPs in *Vkorc1* gene are found at codons 128 (L128S) and 139 (Y139C) on exon 3.^19^ Both L128S and Y139C have been confirmed to confer resistance to ARs using blood clotting tests or non‐choice feeding assays.^16^, ^20^ A combination of mutations in exon 1 and 2, including R12W, A26S, E37E, A48T, and R61L, also known as the *spretus* genotype (*Vkorc1* ^*spr*^), has also been found to confer resistance to FGARs.^21^ In Norway rats, nsSNPs at codon 139 (Y139C, Y139S, Y139F) are the most prevalent documented mutations, followed by codons 120 (L120Q) and 128 (L128Q).^22^ All of these mutations are associated with anticoagulant resistance.^15^, ^16^

Most studies of *Vkorc1* mutations in house mice and Norway rats are from Europe, and research in the U.S. remains limited. In 2009, Rost *et al*.^16^ documented the first *Vkorc1* mutations in the U.S. in a study of Norway rats collected from Illinois and California. Since then, Díaz and Kohn^23^ conducted a survey of *Vkorc1* mutations in three rodent species (house mice, Norway rats, and roof rats) collected across a broad range of the U.S. The authors identified multiple mutations associated with AR resistance with varying frequencies among species and suggested that resistance likely arose through both convergent evolution and gene flow between U.S. and European rodent populations. While this study broadened our understanding of AR resistance, it left geographic gaps, including major metropolitan areas. For instance, Norway rats were not sampled in New Jersey, Pennsylvania, or District of Columbia. Furthermore, the study did not provide information regarding the usage history of ARs, which limits the interpretation of the resistance findings.

In a more recent study, Voit and Richardson^24^ found that no *Vkorc1* mutations were identified in their survey of Virginia rat populations (*n* = 80); however, their sequencing was restricted to exon 3, omitting exons 1 and 2. While previous studies have furthered our understanding of the distribution of *Vkorc1* mutations in the U.S., it is clear that additional studies are needed. Without a comprehensive *Vkorc1* mutation profile, the prevalence of *Vkorc1*‐mutated alleles in wild rodent populations may be underestimated, thereby hindering the development of effective rodent management strategies.

To provide updated information on the distribution patterns of *Vkorc1* mutations, we sampled house mice and Norway rats in the northeastern U.S. and investigated *Vkorc1* SNPs. When available, the rodenticide use history at the collection sites was used to provide additional insight into AR resistance.

## 2 MATERIALS AND METHODS

### 2.1 Study area and sample collection

Pest control companies located in the northeastern U.S., including New York (NY), New Jersey (NJ), Pennsylvania (PA), and Washington, DC, were contacted for rodent sample collection. Those interested in collaboration were provided with a collection kit including instructions, sterile plastic centrifuge tubes (Membrane Solutions LLC, Auburn, WA, USA), scissors, labels, and a pen. After the PMPs found a rodent during their routine rodent control services, they were instructed to clip either the tail (~ 1 in. length) or one ear from the carcass and place it in a tube containing 95% ethanol. The specimens were mailed to Rutgers University and saved at −20 °C until genomic DNA extraction. The species name, collection habitat, and rodenticide use history were provided by the contributing PMPs. Mouse specimens collected in New Brunswick, Paterson, and Trenton in NJ in a previous study were also included^25^ (Rutgers University Institutional Animal Care and Use Committee (IACUC) Protocol No. PROTO202000189). In addition, liver tissue samples were included from house mice collected from NY, NJ, and PA, with the approval of the Drexel University IACUC (Protocol No. LA‐122‐74, Phifer‐Rixey) and the Monmouth University IACUC (Protocol No. Asp1802, Phifer‐Rixey). Rat samples were also collected by the Pest Management team of the New York City Housing Authority or by our laboratory staff following the Rutgers University IACUC Protocol No. PROTO202400033. Most sampling sites were located in residential areas within urban environments (Supporting Information, Table S1). These include three major U.S. metropolitan areas: New York City (NYC) (Four boroughs: Manhattan, Bronx, Brooklyn, Queens), Philadelphia, and Washington, DC. All rodents were collected between July 2021 and July 2025.

### 2.2 DNA extraction, *Vkorc1* gene amplification, and Sanger sequencing

DNA was extracted either from tail or ear clippings or liver tissue. For ear and tail clips, hair and skin were carefully removed from each clip before extracting the DNA to increase the quality of DNA extracts.^26^ Genomic DNA was individually extracted from tail or ear specimens using Qiagen DNeasy Blood and Tissue Kit (Qiagen LLC, Germantown, MD, USA) following the manufacturer's protocol. For liver tissue, genomic DNA was extracted using the Gentra Puregene Tissue Kit as per manufacturer directions (Qiagen LLC, Germantown, MD, USA). All PCR reactions were conducted in a 25 μL volume containing 1 μL of DNA template (80–90 ng/μL) using Taq DNA Polymerase with Standard Taq Buffer (New England Biolabs, Ipswich, MA, USA) following the manufacturer's protocol.

Mitochondrial cytochrome oxidase subunit I (*COI*) was used to confirm the rodent species identification. The *COI* gene was amplified using primers of BatL5310 and R6036R.^27^ The cycle condition for the *COI* gene was 95 °C for 30 s; 35 cycles of 95 °C for 30 s, 54 °C for 40 s, 68 °C for 1 min, and a final extension at 68 °C for 5 min.

For house mice and Norway rats, three coding exons of *Vkorc1* were amplified using different primer sets (Table 1).^23^, ^26^, ^28^, ^29^ For house mice, primers of three exons were additionally attached with universal primers of M13F and M13R to enable DNA sequencing.^23^, ^29^ The cycle conditions for three exons were 95 °C for 30 s; 35 cycles of 95 °C for 30 s, 56 °C for 40 s, 68 °C for 1 min, and a final extension at 68 °C for 5 min. For the Norway rat, the cycle conditions for exons 1 and 3 were 95 °C for 30 s; 31 cycles of 95 °C for 30 s, 54 °C for 40 s, 68 °C for 1 min, and a final extension at 68 °C for 5 min. The annealing temperature for exon 2 was increased to 56 °C.

**Table 1:** Primer sets (forward and reverse) used for amplification and sequencing of three *Vkorc1* exons in house mice and Norway rats

Species	Exon	Primer (5′ – 3′)	Tm (°C)	Length (bp)	References
House mouse*	1	TGCAGCCTCTCCAACTACAAT	56	~900	Díaz and Kohn 2021,^23^ Song *et al*. 2008^29^
		ATGTGCCACCTCACAAACAA			
	2	CGTTCGGGAGTTGAGTCTCT	56	~950	
		ACCTACCAGGTGTGGTCCAA			
	3	GTGCTGGGATTAAAGCATGG	56	~920	
		GAAAGACTGACACCCCGAAG			
Norway rat	1	TAGCTGTCACGCCTAAGAA	54	~900	Díaz and Kohn 2021,^23^ Mooney *et al*. 2018^28^
		GCAAATAAGTGCCTGC			
	2	GGGTGGCGCTTCTTGCTAA	56	~350	
		ACTCCTGCTAAGTGTTCTCCTTG			
	3	CAGGGTTTCTCTGTGTAAC	54	~900	
		CAGACTTGACCAACATAGAA			

* All primer sets for *Vkorc1* exons in house mice were attached with universal primers M13F and M13R to enable DNA sequencing. M13F (5′ – 3′): CACGACGTTGTAAAACGAC; M13R (5′ – 3′): GGATAACAATTTCACACAGG.

PCR products were visualized on a 1% agarose gel and then purified with ExoSAP‐IT Express (Thermo Fisher Scientific Inc., Waltham, MA, USA). Purified PCR amplicons were Sanger sequenced in both forward and reverse directions at Genewiz (Azenta Life Sciences, South Plainfield, NJ, USA).

### 2.3 Data analysis

The sequencing chromatograms were proofread using SnapGene software (www.snapgene.com). Species identity was confirmed by the Basic Local Alignment Search Tool (BLAST) on the NCBI website (NCBI, www.ncbi.nlm.nih.gov). The *Vkorc1* sequence results from house mice were aligned with the reference (NCBI: NM_178600.2); sequences from Norway rats were aligned with the reference (NCBI: NM_203335.2) using the software MEGA 11.^30^ The presence of mutations in three exons was visually inspected and recorded. Heterozygotes were identified by overlapping peaks at the respective nucleotide positions. When one of the forward or reverse sequences for each exon was not successful, the results of the successful amplification were included.

Mutant frequency was calculated as the number of individuals carrying at least one SNP (both homozygous and heterozygous genotypes) divided by the total number of tested rodents, i.e., both homozygotes and heterozygotes were counted as one mutant. Chi‐square tests were conducted to compare mutation frequencies between house mice and Norway rats using R version 4.3.1.^31^

## 3 RESULTS

A total of 305 samples were collected. Among these, eight specimens were too decomposed for DNA extraction and were excluded. Four specimens initially labeled as house mice by PMPs were identified as white‐footed mice (*Peromyscus leucopus* Rafinesque) by *COI* analysis. In addition, exon 3 could not be sequenced from three mice (two from NY and one from NJ). After excluding the abovementioned samples, a total of 147 house mice (*M. m. domesticus*) and 143 Norway rats (*R. norvegicus*) were included in the *Vkorc1* mutation analysis in this study (Supporting Information, Table S1).

### 3.1 *Vkorc1* polymorphisms in house mice

Among the 147 house mice analyzed, 34 were collected from 20 sites (each site was defined as a unique address) across 14 zip codes in three cities (including three NYC boroughs) in NY; 69 were collected from nine sites across seven zip codes in six cities in NJ; and 44 were collected from 24 sites across 23 zip codes in nine cities in PA (Table 2).

**Table 2:** Prevalence of polymorphisms in three exons of *Vkorc1* in house mice collected from New York, New Jersey, and Pennsylvania

State	City‐Borough	No. zip codes (sites*)	No. samples	Wild type	Exon 1		Exon 2		Exon 3						No. mice w/ two mutations
					A32V		W59L		L128S		Y139C		Y139F		
					Hom^†^	Het	Hom	Het	Hom	Het	Hom	Het	Hom	Het	
NY	NYC‐Bronx	1 (1)	12	2	0	0	0	0	1	4	3	5	0	0	3
	NYC‐Brooklyn	1 (1)	1	0	0	0	0	0	1	0	0	0	0	0	0
	Goshen	1 (1)	3	0	0	0	0	1	0	2	0	1	0	0	1
	NYC‐Manhattan	10 (16)	17	0	0	0	4	1	5	5	1	3	1	3	6
	Syracuse	1 (1)	1	0	0	0	1	0	0	0	0	0	0	0	0
	**Subtotal (frequency%)**		**34**	**2 (6%)**	**0 (0%)**		**7 (21%)**		**18 (53%)**		**13 (38%)**		**4 (12%)**		**10 (29%)**
NJ	Branchville	1 (1)	1	1	0	0	0	0	0	0	0	0	0	0	0
	New Brunswick	1 (2)	20	2	0	0	6	2	0	0	10	0	0	0	0
	Newton	1 (1)	2	2	0	0	0	0	0	0	0	0	0	0	0
	Paterson	2 (3)	21	0	0	0	1	0	1	4	13	6	0	0	4
	Runnemede	1 (1)	5	5	0	0	0	0	0	0	0	0	0	0	0
	Trenton	1 (1)	20	0	0	0	8	10	0	0	2	10	0	0	10
	**Subtotal (frequency%)**		**69**	**10 (15%)**	**0 (0%)**		**27 (39%)**		**5 (7%)**		**41 (59%)**		**0 (0%)**		**14 (20%)**
PA	Bryn Mawr	1 (1)	1	1	0	0	0	0	0	0	0	0	0	0	0
	Furlong	1 (1)	1	1	0	0	0	0	0	0	0	0	0	0	0
	Lansdale	1 (1)	1	0	0	0	0	1	0	0	0	0	0	0	0
	Levittown	1 (1)	5	4	0	0	0	0	1	0	0	0	0	0	0
	Middleburg	1 (1)	5	0	0	0	0	0	0	0	4	1	0	0	0
	New Hope	1 (1)	1	0	0	0	0	0	0	1	0	0	0	0	0
	Perkasie	1 (1)	1	1	0	0	0	0	0	0	0	0	0	0	0
	Philadelphia	15 (16)	24	0	0	2	0	2	18	4	1	2	0	0	5
	Reading	1 (1)	5	4	0	0	0	0	0	1	0	0	0	0	0
	**Subtotal (frequency%)**		**44**	**11 (25%)**	**2 (5%)**		**3 (7%)**		**25 (59%)**		**8 (18%)**		**0 (0%)**		**5 (11%)**
**Total (frequency%)**			**147**	**23 (16%)**	**2 (1%)**		**37 (25%)**		**48 (33%)**		**62 (42%)**		**4 (3%)**		**29 (20%)**

* A site refers to a unique address.

^†^ Hom and Het indicate homozygous and heterozygous mutations, respectively.

**Figure 1:**
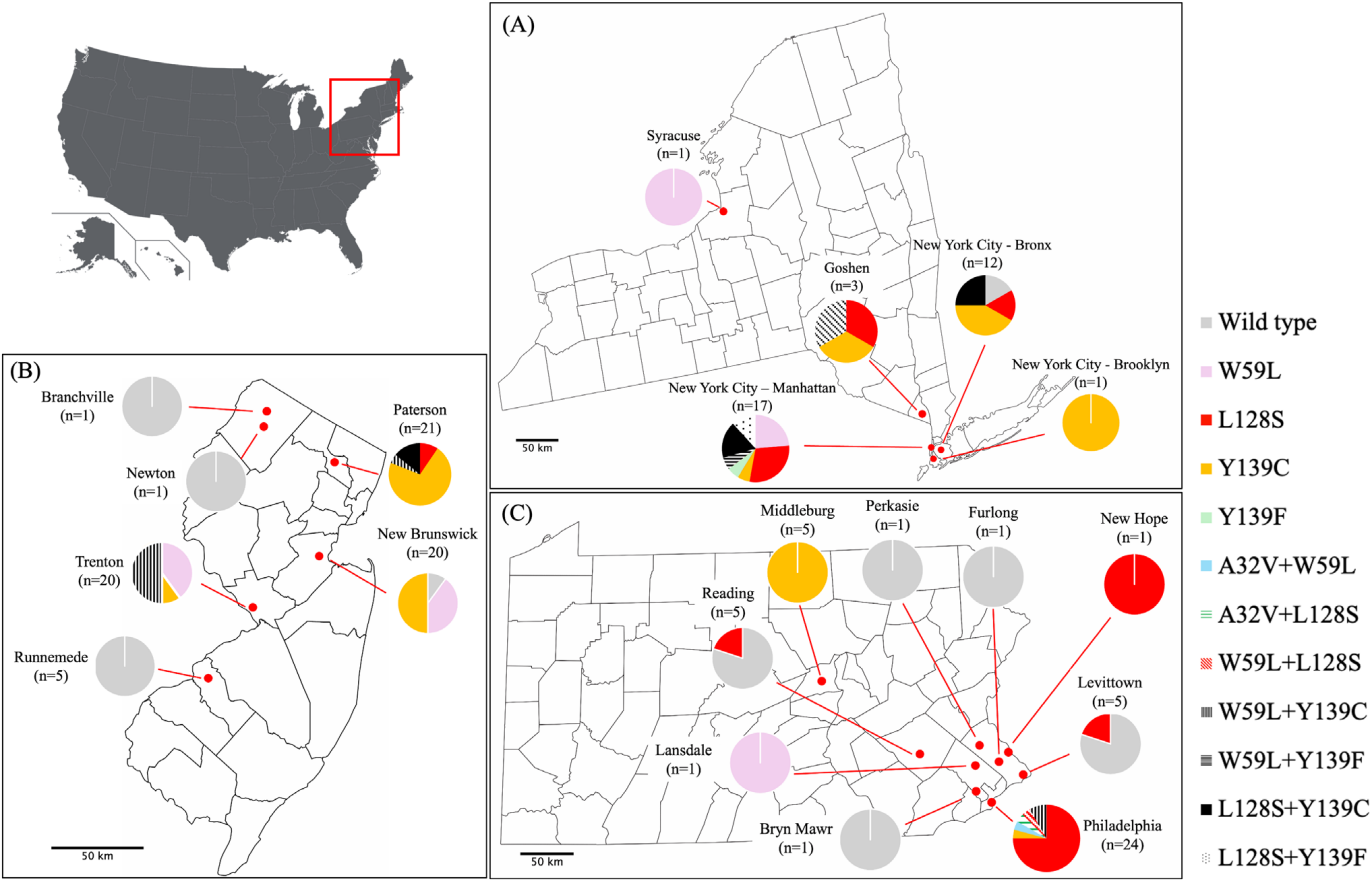
Prevalence of *Vkorc1* polymorphisms in house mice collected from (A) New York, (B) New Jersey, and (C) Pennsylvania.

Five nsSNPs were detected in the *Vkorc1* gene in our survey: A32V in exon 1, W59L in exon 2, and L128S, Y139C, and Y139F in exon 3 (Fig. 1; Table 2). No sSNPs were detected. Notably, in this study, the A32V mutation is reported for the first time in house mice and the Y139F mutation for the first time in *M. m. domesticus*. Both mutations were rare. The A32V mutation was found in only two heterozygous mice in Philadelphia, PA (Table 2) and, in both cases, in combination with another nsSNP (W59L or L128S; Table 3 and Fig. 1). The Y139F mutation was found in only four mice in Manhattan, NY (one homozygote and three heterozygotes; Table 2).

**Table 3:** Number of combinations of *Vkorc1* polymorphisms in house mice collected from New York, New Jersey, and Pennsylvania*

State	City‐Borough	Combination genotype						
		A32V + W59L	A32V + L128S	W59L + L128S	W59L + Y139C	W59L + Y139F	L128S + Y139C	L128S + Y139F
NY	NYC‐Bronx	0	0	0	0	0	3	0
	Goshen	0	0	1	0	0	0	0
	NYC‐Manhattan	0	0	0	0	1	3	2
NJ	Paterson	0	0	0	1	0	3	0
	Trenton	0	0	0	10	0	0	0
PA	Philadelphia	1	1	1	2	0	0	0

* Each nsSNP among all mutation combinations was heterozygous.

Among all mice across the three states, Y139C was the most common mutation, with a frequency of 42%, followed by L128S (33%) and W59L (25%). However, the distribution of nsSNPs differed across the sampled areas (Fig. 1). The Y139C mutation was detected in 62 total mice (34 homozygotes, 28 heterozygotes) with the highest frequency observed in NJ (59%), followed by NY (38%) and PA (18%) (Table 2). The L128S mutation was detected in 48 mice (27 homozygotes, 21 heterozygotes). It was the predominant mutation among the five nsSNPs in NY and PA, with frequencies of 53% and 59%, respectively. The W59L mutation was detected in 37 mice (20 homozygotes, 17 heterozygotes) from three states, with the highest frequency observed in NJ (39%), followed by NY (21%) and PA (7%; Table 2).

While no mice were found to carry more than two mutations in *Vkorc1*, mice carrying two mutations were common (~20% of mice (29 out of 147) across five cities, including NYC (NYC‐Bronx, NYC‐Manhattan), Goshen, Paterson, Trenton, and Philadelphia; Table 3). The observed mutation combinations were A32V + W59L, A32V + L128S, W59L + L128S, W59L + Y139C, W59L + Y139F, L128S + Y139C, and L128S + Y139F (Fig. 1, Table 3). Notably, both nsSNPs in mice that harbored two mutations were heterozygous.

### 3.2 *Vkorc1* polymorphisms in Norway rats

Among the 143 Norway rats analyzed, 78 were collected from seven sites across six zip codes in four boroughs of NYC; six were collected from two sites across two zip codes in two cities in NJ; 32 were collected from 12 sites across five zip codes in two cities in PA; and 27 were collected from seven sites across six zip codes in Washington, DC (Table 4).

**Table 4:** Prevalence of polymorphism in two exons of *Vkorc1* in Norway rats collected from New York, New Jersey, Pennsylvania, and District of Columbia

State	City‐Borough	No. zip codes (sites*)	No. samples	Wild type	Exon 2				Exon 3	
					H68H		I82I		L128V	
					Hom^†^	Het	Hom	Het	Hom	Het
NY	NYC‐Bronx	2 (3)	42	20	0	0	7	15	0	0
	NYC‐Brooklyn	1 (1)	15	11	0	0	1	0	0	3
	NYC‐Manhattan	2 (2)	9	3	0	0	2	4	0	0
	NYC‐Queens	1 (1)	12	8	1	1	0	2	0	0
	**Subtotal (frequency%)**		**78**	**42 (54%)**	**2 (3%)**		**31 (40%)**		**3 (4%)**	
NJ	Jersey City	1 (1)	5	5	0	0	0	0	0	0
	New Brunswick	1 (1)	1	1	0	0	0	0	0	0
	**Subtotal (frequency%)**		**6**	**6 (100%)**	**0 (0%)**		**0 (0%)**		**0 (0%)**	
PA	Philadelphia	4 (11)	12	8	0	0	2	2	0	0
	Pittsburgh	1 (1)	20	20	0	0	0	0	0	0
	**Subtotal (frequency%)**		**32**	**28 (88%)**	**0 (0%)**		**4 (12%)**		**0 (0%)**	
Washington, DC	Washington, DC	6 (7)	27	17	0	0	4	6	0	0
	**Subtotal (frequency%)**		**27**	**17 (63%)**	**0 (0%)**		**10 (37%)**		**0 (0%)**	
**Total (frequency%)**			**143**	**93 (65%)**	**2 (1%)**		**45 (32%)**		**3 (2%)**	

* A site refers to a unique address.

^†^ Hom and Het indicate homozygous and heterozygous mutations, respectively.

**Figure 2:**
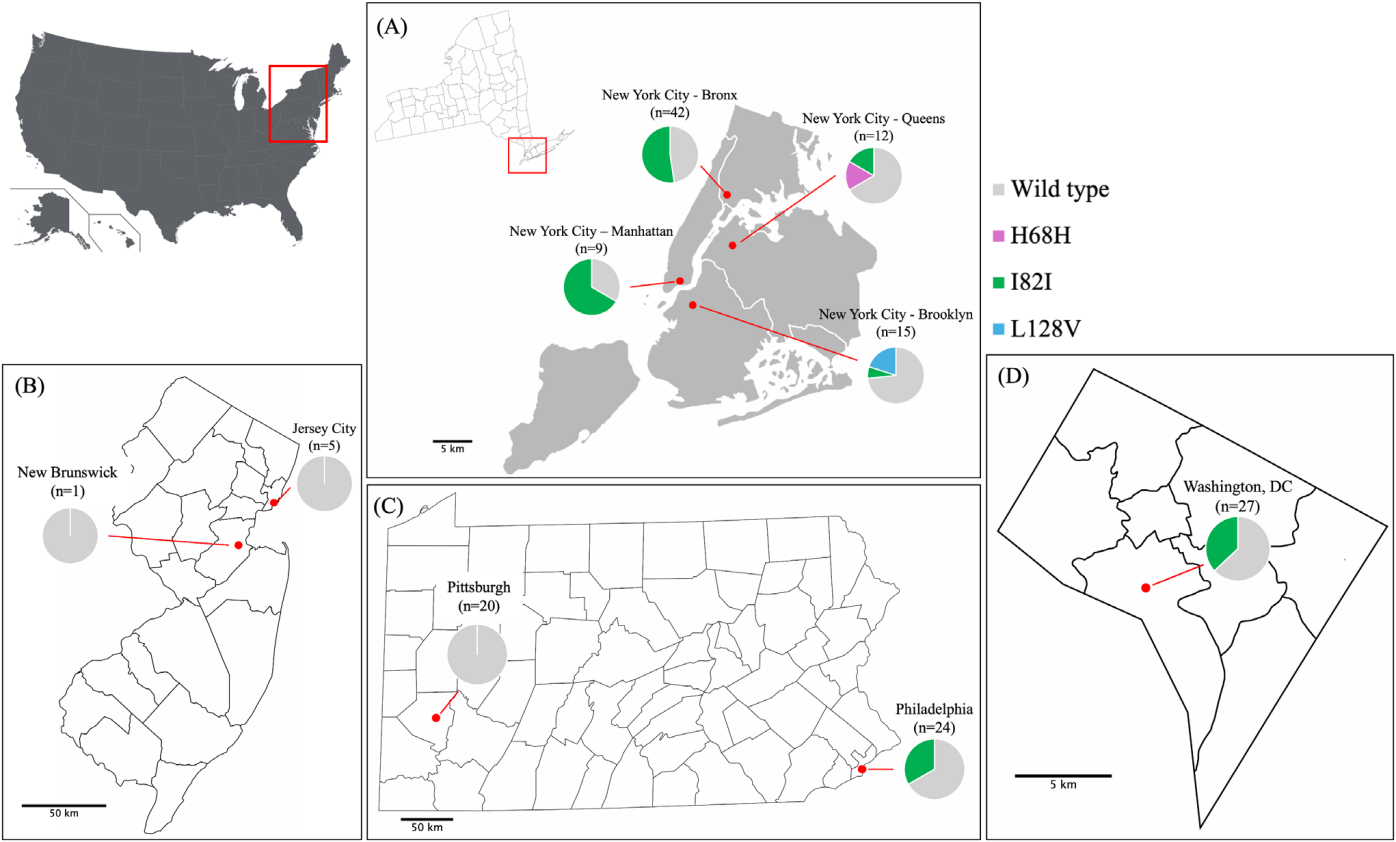
Prevalence of *Vkorc1* polymorphisms in Norway rats collected from (A) New York, (B) New Jersey, (C) Pennsylvania, and (D) Washington, DC.

Three mutations were identified, including two sSNPs (H68H and I82I) in exon 2 and one nsSNP (L128V) in exon 3. The distribution of SNPs in rats varied among cities and different boroughs within NYC (Fig. 2). The H68H mutation was detected only in two rats collected from NYC‐Queens, of which one was homozygous (Table 4). Among Norway rats across all states, the I82I mutation was the most common mutation, with a frequency of 32% (45 out of 143;16 homozygotes, 29 heterozygotes; Table 4). Moreover, the I82I mutation was the predominant mutation in NY, PA, and Washington, DC, with frequencies of 40%, 12%, and 37%, respectively. The L128V mutation has not previously been detected in Norway rats and was detected only in NYC‐Brooklyn, where three heterozygous individuals were identified (Table 4). No rats were observed carrying more than one mutation simultaneously.

In total, house mice had a significantly higher frequency of mutants (counted as individuals with at least one nsSNP *Vkorc1* mutation) compared to Norway rats (χ ^2^ = 193.01, df = 1, *P* < 0.001), with frequencies of 84% and 2%, respectively (Tables 2 and 4).

## 4 DISCUSSION

Monitoring the prevalence of resistance to ARs is essential for improving strategies of rodent management and understanding the impacts of ARs on the environment. In this study, we screened SNPs in the *Vkorc1* gene of house mice and Norway rats collected from urban environments in the northeastern U.S. A total of five nsSNPs were identified in house mice, and two sSNPs and one nsSNP were detected in Norway rats. Overall, mutations in *Vkorc1* were common in both species, but while genotypes known to be associated with resistance were common in house mice, they were not observed in Norway rats. Importantly, this study complements previous work^23^ by addressing data gaps in major cities and provides up‐to‐date information on the occurrence of SNPs in the *Vkorc1* gene for the sampled regions in the U.S. In addition, to our knowledge, in this study, the A32V mutation is reported for the first time in house mice, the Y139F mutation for the first time in *M. m. domesticus*, and the L128V mutation for the first time in Norway rats.

### 4.1 *Vkorc1* polymorphisms in house mice

Among multiple *Vkorc1* polymorphisms reported in house mice, three genotypes have been demonstrated to confer resistance to ARs and have been found to be prevalent in European house mouse populations: *Vkorc1* ^*spr*^, L128S, and Y139C.^18^, ^19^, ^28^, ^32^, ^33^ In the U.S., the previously observed *Vkorc1* polymorphisms in mice were: A21T, E37E in exon 1, W59L in exon 2, and I104V, I123I, V118L, L128S, Y139C in exon 3.^23^ While our survey also documented W59L in exon 2 and L128S and Y139C in exon 3, we newly uncovered the presence of A32V in exon 1 and Y139F in exon 3 (Table 2).

The A32V mutation in exon 1 was rare (Table 2). Because it is novel, there are no known associations with AR resistance and studies testing the impact of A32V on AR resistance are needed. The Y139F mutation in exon 3 was also rare, detected in just four samples and restricted to Manhattan. Previously, the Y139F mutation was found in 80.2% of the *Mus m. musculus* samples in the Czech Republic.^34^ The authors exposed *M. m. musculus* carrying homozygous Y139F to a SGAR (bromadiolone) in choice feeding tests, observing 100% survival after 21 days. This data suggests that the Y139F mutation is likely to confer SGAR resistance in this close relative of *M. m. domesticus*. Interestingly, a previous survey of 87 mice in NYC‐Manhattan^23^ did not identify any mice with this mutation. Further studies with larger sample sizes from a wider sampling area are needed to better understand the origin and expansion of the Y139F mutation among *M. m. domesticus*.

We found that the W59L mutation in exon 2 was commonly detected in eight cities with a frequency of 25% (Table 2). This mutation appears widespread and has been reported previously in Germany^18^ and in five U.S. cities.^23^ Nevertheless, its impact on AR resistance has not been tested using either *in vivo* tests or biokinetic simulations.^19^ The high prevalence of this mutation provides strong motivation for tests of how, if at all, it might affect AR resistance.

In exon 3, mice carrying the L128S mutation^15^, ^20^, ^28^, ^35^ and the Y139C mutation^16^, ^20^, ^28^, ^35^, ^36^ have been confirmed to show resistance to FGARs and SGARs. In the U.S., L128S and Y139C were previously reported at frequencies of 10% and 23%, respectively, among 556 house mice sampled across 24 states.^23^ In comparison, our study found higher frequencies of L128S (33%) and Y139C (42%) among 147 mice from three states (Table 2). While the sampling methods differ between the studies, both provide strong evidence that these variants (L128S and Y139C) that are known to confer AR resistance are common in the U.S.

Mice carrying multiple SNPs in the *Vkorc1* gene have been reported in several studies.^19^, ^20^, ^23^, ^35^, ^37^, ^38^ In this study, seven combinations of two *Vkorc1* mutations were detected in 29 mice (Table 3). Among the seven combinations, the L128S + Y139C has been found to have similar resistance to brodifacoum as the variant Y139C,^36^ whereas the other six combinations are novel. Four combinations, A32V + L128S, W59L + L128S, W59L + Y139C, and L128S + Y139F, contain one known resistance‐associated mutation (L128S or Y139C). However, it is not known whether multiple mutations interact to impact resistance in these cases and thus the functional significance of these combinations remains unclear.

### 4.2 *Vkorc1* polymorphisms in Norway rats

In the U.S., nine SNPs have previously been reported in Norway rats, including R12R, R35P, G46S, I82I, I90L, L94L, I107I, T137T, and A143A.^16^, ^23^ In the current study, we identified three SNPs (H68H, I82I, and L128V), of which H68H was identified for the first time in the U.S. and L128V was identified for the first time in any population.

The H68H mutation has previously been found in Hong Kong,^39^ China,^22^, ^40^ and Australia^41^ but blood clotting response tests suggest that it is not associated with warfarin resistance.^40^ The I82I mutation is also widespread and has been detected in several countries in Europe,^16^, ^42^ China,^22^, ^40^ and Australia^41^; however, its contribution to AR resistance remains untested.

In exon 3, nsSNPs at codons 120, 128, and 139 have been commonly reported to confer ARs resistance in rats,^15^, ^16^, ^32^, ^42^, ^43^, ^44^ but no mutations in these codons have previously been detected in the U.S. In the current study, we identified L128V (Leucine to Valine) from three samples collected from NYC‐Brooklyn. Rat infestations at this collecting site were treated with both FGAR and SGAR (chlorophacinone and difethialone), as well as other non‐chemical methods, such as snap traps (Supporting Information, Table S1). Other mutations at this codon, L128Q^17^, ^44^ and L128S,^42^ have previously been detected in Norway rats in other countries. Given that other mutations at codon 128 have been shown to confer warfarin resistance,^15^ this new mutation at codon 128 should be investigated for anticoagulant resistance.

L128V was the only nsSNP identified in rats in the current study. When combined with the three nsSNPs previously reported,^16^, ^23^ four nsSNPs have now been identified in U.S. Norway rats to date. The total number of nsSNPs documented in *Vkorc1* in Norway rats is significantly lower in the U.S. than in Europe, where 23 nsSNPs have been detected.^22^, ^38^ Similarly, Norway rat populations in Australia and New Zealand have low reported nsSNP diversity.^41^, ^45^ One explanation for the lower number of nsSNPs in Norway rat populations from the U.S. and other regions colonized from Europe is the founder effect, which can reduce genetic diversity genome‐wide. In the current study, rats were collected from the northeastern U.S., where rat populations are genetically derived from western European populations.^46^, ^47^ The low number of nsSNPs may reflect limited variation in the *Vkorc1* gene among the original founding European ancestors. However, it is noteworthy that rats from NYC, Philadelphia, and Washington, DC did not exhibit evidence of the diverse *Vkorc1* genotypes observed in European populations despite likely frequent opportunities for transatlantic exchange *via* economic activities. This result implies limited effective gene flow among U.S. and European Norway rat populations. Additional sampling and genomic data are needed to better understand the origins and spread of *Vkorc1* mutations in the U.S.

### 4.3 Dynamics of *Vkorc1* polymorphisms in mice and rats and the implications for rodent management and environmental impacts

Compared with the previous survey conducted by Díaz and Kohn,^23^ we observed a higher occurrence of *Vkorc1* SNPs in house mice (84% *vs* 33%). In addition, while Díaz and Kohn^23^ documented a resistance frequency of 29% in house mice (i.e., the presence of L128S and Y139C mutations), we estimated a resistance frequency of 69% (111 mice carrying either L128S or Y139C or both out of 147 total; Tables 2 and 3). For Norway rats, the occurrence of *Vkorc1* mutations was similar (35% in our study *vs* 43%). However, none of the individuals sampled in our study carried mutations known to confer resistance, whereas Díaz and Kohn^23^ reported a resistance frequency of 7%. While the differences in results among the studies for house mice are striking, it is important to note that the two studies differ markedly in geographic coverage and sampling design. For instance, Díaz and Kohn^23^ sampled house mice (*n* = 11) from only one location in NJ with no records of AR use. In contrast, our study sampled mice (*n* = 69) from nine sites in NJ, where most (six out of nine) sites reported the use of AR baits, which may contribute to the higher prevalence of *Vkorc1* mutations. Because of the differences in sampling between the studies, it is not possible to determine if AR resistance has increased over time. However, the high prevalence of AR resistance in house mice in both studies provides strong justification for consistent, long‐term monitoring of *Vkorc1* mutations in diverse house mouse and Norway rat populations to better understand the dynamics of rodenticide resistance and improve management of rodent populations.

In our study, we observed that mice had significantly higher (84%) nsSNP mutant frequency than Norway rats (2%). The much higher frequency of *Vkorc1* mutation in mice compared to rats has also been reported in Europe.^28^, ^48^ This difference between rats and mice has been attributed to the complex interplay of rodenticide exposure, genetic diversity, and behavioral and habitat preferences.^49^ Apart from habitat differences, house mice are often characterized as neophilic (attracted to novel objects),^50^, ^51^ whereas rats exhibit neophobic tendencies (showed wariness and avoidance of novel objects).^52^, ^53^ In some cases, rats have evolved heightened neophobic behaviors in response to intensive rodent control efforts, such as avoidance of bait containers.^54^ As a result, rats may have a lower tendency to approach and consume AR baits than house mice, which could translate into differences in selective pressures acting on the *Vkorc1* locus. However, bait consumption is only one factor out of many that potentially contributes to the observed disparity in mutation frequencies. Further research is needed to better understand *Vkorc1* mutation dynamics. Moreover, beyond the mutations in *Vkorc1*, other mechanisms, such as overexpression of P450 genes and behavioral avoidance, can also contribute to resistance to ARs in rodents.^5^, ^55^ For instance, studies have found that warfarin resistance increased in Norway rats without mutations in *Vkorc1*.^15^, ^44^, ^56^ While surveying *Vkorc1* mutations provides valuable insights, complementary *in vivo* and *in vitro* assays, as well as biokinetic simulations, are necessary for a comprehensive assessment of resistance to ARs in rodent populations.

In the current study, all of the mouse and rat sampling sites with known treatment histories deployed AR baits, with difethialone being the most frequently used active ingredient (Supporting Information, Table S1). Among house mouse samples, 11 of the 13 sites with known treatment histories used difethialone; for rat samples, eight of the nine sites with known treatment histories used difethialone. Based on the high frequency of resistance‐associated genotypes detected in house mice in this study, reliance on ARs alone may not provide effective control and exacerbate the development of resistance. Furthermore, non‐target animals have been shown to be threatened by ARs through primary and secondary poisoning.^57^, ^58^ SGARs are more persistent than FGARs and are more concerning for non‐target species due to their biomagnification effect.^59^ In the U.S., ARs continue to be detected in a variety of wildlife species in alarming concentrations, including birds,^59^, ^60^, ^61^ fishers,^62^ and common urban rodent predators (raccoon, skunk, and opossum)^63^ despite regulations requiring rodenticide baits to be placed in tamper‐resistant stations and can only be applied by licensed professionals (U.S. EPA, https://www.epa.gov/rodenticides/restrictions-rodenticide-products#types). Given the hazards to non‐target wildlife and environments, it is essential to educate PMPs and the public on the proper use of ARs and to safeguard wild animals. The use of non‐anticoagulants, such as bromethalin and cholecalciferol, should also be considered when rodenticides are needed. Integrating multiple methods including exclusion, sanitation, habitat modification, and trapping, will be critical for reducing reliance on ARs and achieving more sustainable and effective rodent control.

## 5 CONCLUSION

This study documented the prevalence of SNPs in the *Vkorc1* gene in house mice and Norway rats in the northeastern U.S. We found that a high percentage (84%) of house mice sampled had at least one *Vkorc1* mutation and 20% of tested mice had two *Vkorc1* mutations. Notably, 69% of the mice carried mutations that are known to confer AR resistance. In Norway rats, we found that a lower percentage (35%) of individuals had mutations in the *Vkorc1* gene, with two sSNPs and one nsSNP observed. However, one of these mutations does not confer AR resistance and the contribution of the other two to AR resistance remains uninvestigated. Overall, our survey suggests that *Vkorc1* diversity and prevalence in house mice and Norway rats differ markedly between Europe and the U.S., with higher diversity in Europe. For example, the *Vkorc1* ^*spr*^ genotype has been widely reported in European house mouse populations but was absent in the U.S. and the number of nsSNPs reported in Norway rats is much lower in the U.S. These results indicate that the demographic effects of their colonization history and local selection pressures may be shaping resistance profiles more than ongoing gene flow between U.S. and European populations. Further evidence is needed to clarify the origin and spread of *Vkorc1* mutations in U.S. rodent populations. Importantly, we provide strong evidence that AR resistance conferred *via* mutations in *Vkorc1* is common in house mice, thus suggesting that management strategies should reduce reliance on ARs to ensure effective control.

## CONFLICT OF INTEREST

The authors declare that they have no known competing financial interests or personal relationships influencing the results reported in this study.

## Supporting information


**Table S1.** The information of house mice collected from the U.S. in this study.

## Data Availability

The data that support the findings of this study are available from the corresponding author upon reasonable request.
